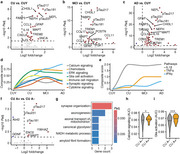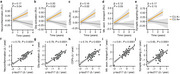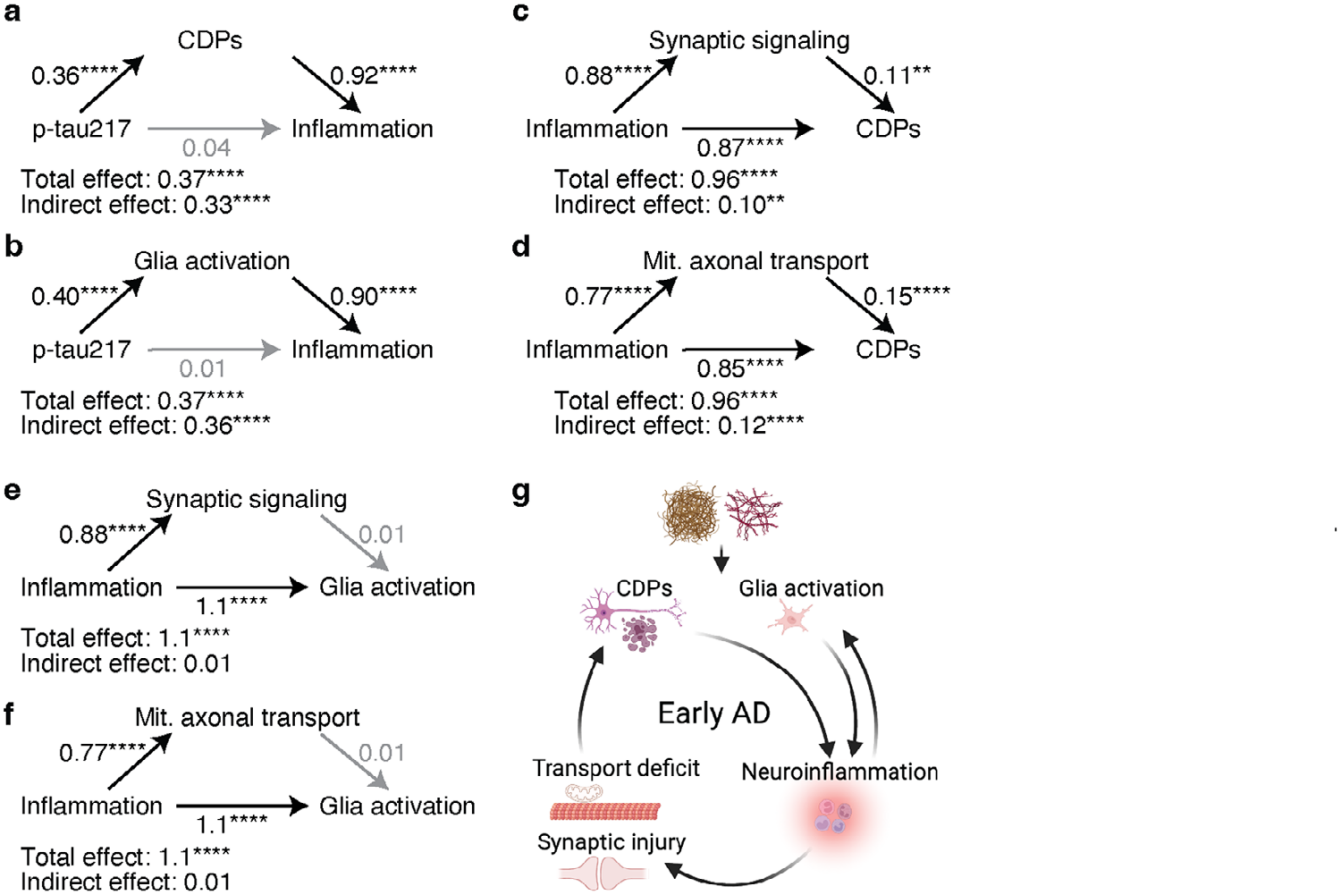# Astrocyte and Glial interaction in developing novel therapeutic strategies

**DOI:** 10.1002/alz70856_103468

**Published:** 2025-12-26

**Authors:** Marcel Seungsu Woo, Joseph Therriault, Yi‐Ting Wang, Seyyed Ali Hosseini, Arthur C. Macedo, Nesrine Rahmouni, Etienne Aumont, Stijn Servaes, Cécile Tissot, Jaime Fernandez Arias, Lydia Trudel, Brandon J Hall, Gleb Bezgin, Kely Monica Quispialaya Socualaya, Marina P Gonçalves, Tevy Chan, Jenna Stevenson, Yansheng Zheng, Robert Hopewell, Firoza Z Lussier, Gassan Massarweh, Paolo Vitali, Jean‐Paul Soucy, Andrea Benedet, Serge Gauthier, Henrik Zetterberg, Kaj Blennow, Tharick A Pascoal, Pedro Rosa‐Neto

**Affiliations:** ^1^ University Medical Center Hamburg‐Eppendorf, Hamburg, Germany; ^2^ McGill University, Montreal, QC, Canada; ^3^ Montreal Neurological Institute, Montreal, QC, Canada; ^4^ Translational Neuroimaging Laboratory, The McGill University Research Centre for Studies in Aging, Montréal, QC, Canada; ^5^ Lawrence Berkeley National Laboratory, Berkeley, CA, USA; ^6^ University of Pittsburgh, Pittsburgh, PA, USA; ^7^ The McGill University Research Centre for Studies in Aging, Montreal, QC, Canada; ^8^ Montreal Neurological Institute, McGill University, Montréal, QC, Canada; ^9^ Institute of Neuroscienace and Physiology, University of Gothenburg, Mölndal, Västra Götaland, Sweden; ^10^ Institute of Neuroscience and Physiology, Sahlgrenska Academy, University of Gothenburg, Gothenburg, Sweden; ^11^ Department of Psychiatry and Neurochemistry, University of Gothenburg, Gothenburg, Sweden; ^12^ University of Pittsburgh School of Medicine, Pittsburgh, PA, USA

## Abstract

**Background:**

Neuroinflammation is hallmark of Alzheimer's disease (AD) that drives the accumulation of amyloid‐β (Aβ) and neurofibrillary tangles (NFTs). However, the role of neuroinflammation for progression in preclinical AD has not been defined.

**Method:**

We used Nucleic acid Linked Immuno‐Sandwich Assay (NULISA) for targeted proteomics in 32 cognitively unimpaired individuals younger than 30 years (CUY), 154 cognitively unimpaired older than 30 years (CU), 39 people with mild cognitive impairment (pwMCI), 50 pwAD and 107 people with other neurological diseases (OND). Longitudinal data was available for 146 individuals with a mean follow‐up of 26 months. We used unsupervised analyses to identify biological themes and performed cross‐sectional association and mediation analyses with Aβ‐PET, tau‐PET, structural MRI, different blood phospho‐tau (*p*‐tau) analytes as well as longitudinal analyses.

**Results:**

We defined gene ontology themes and pathways that were differently regulated across the AD continuum. We detected that CSF signatures for glia activation, immune signaling, and calcium signaling gradually increased during aging and across the AD continuum. Notably, glia activation and calcium signaling signatures were already impaired in CU A+ in comparison to CU A‐. Further longitudinal analyses in CU revealed an increase of neuroinflammation, glia activation, activation of cell death pathways and deficits in mitochondrial transport in A+ but not in A‐ participants underlining the specificity for AD. This increase was significantly correlated with *p*‐tau217 progression in CU A+. Finally, mediation analyses in A‐T‐ and A+T‐ participants revealed that activation of cell death pathways and glia activation mediated the effect of early AD progression measured by *p*‐tau217 on neuroinflammation. Additionally, we found a significant association between neuroinflammation and activation of cell death pathways and glia activation. However, only the effect on cell death pathway activation but not glia activation was mediated by altered synaptic signaling and disturbed mitochondrial axonal transport.

**Conclusion:**

We show that neuroinflammation and cell death pathways are important drivers of disease progression in preclinical and early AD. We propose that Aβ initiates a vicious cycle of neuroinflammation, glia activation, neuronal dysfunction and activation of cell death pathways that underlines the need for immunomodulatory interventions already in the early AD disease phase.